# RSV infection in children hospitalised with severe lower respiratory tract infection in a low-middle-income setting: A cross-sectional observational study

**DOI:** 10.1371/journal.pone.0291433

**Published:** 2023-09-14

**Authors:** Nicole Morgan, Heloise Buys, Rudzani Muloiwa

**Affiliations:** 1 Department of Paediatrics & Child Health, University of Cape Town, Cape Town, South Africa; 2 Red Cross War Memorial Children’s Hospital, Cape Town, South Africa; University of Buea, CAMEROON

## Abstract

**Introduction:**

Low- and middle-income countries carry the largest burden of Respiratory syncytial virus (RSV) disease, with most deaths occurring in these settings. This study aimed to investigate the burden of RSV disease in South African children hospitalised with lower respiratory tract infection (LRTI), with specific reference to incidence, risk factors, and co-infections.

**Methods:**

A database from a previous prospective study containing demographic, laboratory and clinical data on children hospitalised with LRTIs in Cape Town, South Africa, was used. A nasopharyngeal swab (NP) and induced sputum (IS) were tested for RSV PCR. Descriptive statistics were used to characterise the study population, and a multivariable analysis of risk factors and co-infections was done.

**Results:**

RSV was detected in 142 (30.9%; 95% CI 26.7–35.3) of the included 460 study children with LRTI. The median age of RSV-positive children was 4.6 (IQR 2.4–9.7) months compared to RSV-negative children of 10.5 (IQR 4.4–21.3) months, P = <0.001. Most cases occurred in autumn and winter with 126 (89%) cases over this period. IS demonstrated greater sensitivity for RSV diagnosis with 135 cases (95.1%) detected on IS and 57 cases (40.1%) identified on NP; P<0.001. The median length of hospital stay was 3.3 (SD 4.2) days in the RSV positive group and 2.7 (SD 3.3) days in the RSV negative group; P<0.001. The median number of detected viral pathogens was 1 (IQR 0–2) in RSV-positive children (when RSV was excluded from the count) compared to 2 (IQR 2–3) in RSV negative children; P<0.001. The presence of RSV was independently associated with a reduction in the frequency of most viruses tested for on PCR.

**Conclusions:**

RSV is common in children hospitalised with LRTI and mainly affects younger children. There is an urgent need to find an effective vaccine to prevent RSV pneumonia in children worldwide, especially in LMICs that carry the greatest burden of disease.

## Introduction

Lower respiratory tract infections (LRTIs) are a leading cause of death in children worldwide. Following the implementation of successful vaccination programs, the causative pathogens have changed with viruses emerging to dominate over bacterial causes [1]. The world has seen a substantial reduction in LRTI deaths with mortalities having halved over the last twenty years. However, there is still much progress to be made, specifically in low- and middle-income countries (LMICs) [2]. The incidence of Respiratory syncytial virus (RSV) associated pneumonia is similar globally, but LMICs carry 99% of the burden of RSV disease-related mortality [3].

In addition to mortality, RSV causes significant morbidity in childhood. Almost all of the world’s children will be infected with RSV by their second birthday. Repeated infections may occur, as RSV infection does not seem to confer protective immunity against subsequent infections [4, 5]. A study done in the Western Cape province of South Africa demonstrated that LRTIs in early infancy may be associated with decreased lung function at the age of 1 year [6]. This impaired lung function may persist through childhood into adulthood and if it did, could lead to an increased risk of recurrent respiratory infections and chronic respiratory disease later on in life [7, 8].

Universally, the majority of severe RSV disease occurs in younger children, mostly under the age of one. Although most children admitted to hospital with RSV pneumonia are previously healthy, risk factors for severe disease have been identified [1]. There is an increased risk of severe disease in babies born prematurely and in those with pre-existing cardiopulmonary disease [5]. Malnutrition and HIV, especially relevant in LMICs such as South Africa, have been found to predispose children to severe disease [5, 9]. The 2016 Global Burden of Disease Study, conducted in the same year as our study, found that childhood wasting was present in 60% of deaths associated with LRTIs in the under 5 age group [10].

RSV is estimated to cause 33 million LRTIs per annum and 3.4 million hospitalisations in children under the age of 5 years worldwide [11]. RSV infection occurs in distinct seasonal patterns worldwide. In South Africa, RSV infection shows a predilection for the autumn and winter months [12]. Recent reports have highlighted the role of RSV outbreaks in disrupting health services in both high income and LMIC settings. This has placed a huge burden on health systems and has had significant cost implications for countries around the world [13, 14].

In South Africa, as in most LMICs, RSV infections are rarely confirmed by laboratory diagnosis with diagnoses most often being made clinically, making the burden of RSV disease difficult to assess [15]. However, understanding trends of the burden of RSV disease will assist in prioritising interventions and help track progress towards global goals in both the prevention and treatment of LRTIs [9].

There is currently no specific treatment for RSV disease and management is largely supportive [5]. This places a huge burden on health systems and has significant cost implications for countries around the world. The prevention of severe disease has therefore become a global target strategy. There is currently no registered RSV vaccine available worldwide. Monoclonal antibodies, such as palivizumab, have shown promise in vulnerable children but the dosing logistics are impractical and the costs unaffordable to most LMICs [16].

This study aimed to investigate the burden of RSV disease at a tertiary children’s hospital in Cape Town, South Africa. We aimed to determine the prevalence of PCR confirmed infection, risk factors for severe disease, and associated co-infections over a one year period. Secondarily, the study sought to assess appropriate method of diagnosis. As other studies suggested that IS and NP may have different abilities to identify pathogens, the study sought to secondarily assess the best sample to be used for diagnostic confirmation [17].

## Materials and methods

### Study population

**Fig 1:**
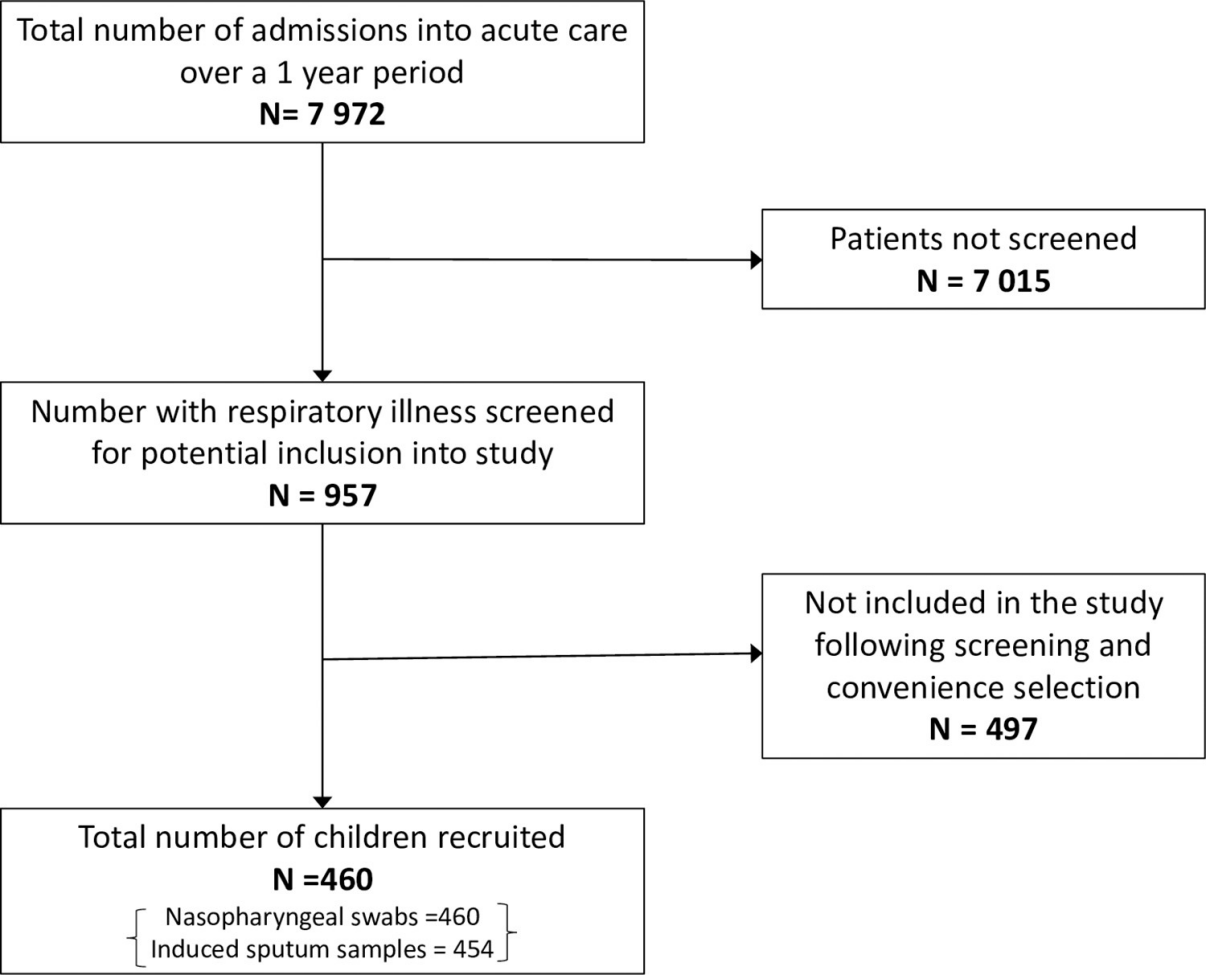
Flow diagram of participants in the study.

The current study is a sub-study of the one previously published in 2016 [17]. It uses existing data collected prospectively over a one-year period between September 2012 and September 2013 at the Red Cross War Memorial Children’s Hospital (RCWMCH) in Cape Town, South Africa. Briefly, the study includes 460 children, aged less than 13 years who were admitted to the short stay paediatric ward with an acute respiratory illness. Inclusion criteria were WHO-defined age-specific tachypnoea or lower chest wall indrawing, apnoea, age less than 13 years, informed parental consent and enrolment into the study within 48 hours of admission. A maximum of 4 children were conveniently enrolled per working day to ensure even distribution of enrolment throughout the year. Participants were excluded if they had a previous admission to a healthcare facility in the preceding two weeks. We used the WHO ‘severe acute respiratory infection’ definition when referring to severe disease—‘an acute respiratory illness with a history of fever or measured fever of ≥ 38°C and cough, with onset within the past 10 days requiring hospitalisation’ [18]. All children were managed according to national and departmental guidelines and were followed up until discharge from hospital **Fig 1.**

### Procedures

The study was conducted at RCWMCH, a public referral hospital catering for children outside the neonatal period aged up to 13 years. Caregivers of all participants were interviewed for clinical information and patient outcomes were obtained from clinical notes. The Road to Health card, an individualised handheld record, was reviewed to provide information regarding birth history, immunization status and growth. The nutritional status of each child was classified according to WHO weight-for-age Z-score criteria as normal (above -1 Z score), mildly (between -1 and -2 Z score)-, moderately (between -2 and -3 Z score)- or severely (less than -3 Z score)- underweight-for-age [19]. History of HIV exposure, infection and antiretroviral therapy (ART) were also noted. All children were tested for HIV. HIV infection was confirmed with an HIV PCR test in children less than 18 months old, or with two ELISA tests in children older than 18 months. Children were classified as ‘HIV infected’—i.e., all children living with confirmed HIV infection; ‘HIV-exposed-uninfected (HEU)’–i.e., all children born to mothers living with HIV infection but who tested negative on laboratory HIV PCR or Elisa testing; or ‘HIV- unexposed-uninfected- (HUU)’- i.e., children born to mothers who had tested negative for HIV, and who themselves tested negative for HIV on HIV PCR or Elisa testing as appropriate for their age.

In order to obtain microbiologic confirmation of RSV infection, a nasopharyngeal swab (NP) and an induced sputum (IS) were sequentially performed. IS was collected after nebulisation with a bronchodilator and hypertonic saline. Specimens were then sent to the laboratory for PCR testing on a multiplex real-time PCR assay, the FTD® Resp 33 (Fast-Track Diagnostics, Esch-sur-Alzette, Luxembourg). The assay is able to identify presence of a range of viruses and bacteria.

### Ethical approval

Ethical approval for this study was granted by the Human Research Ethics Committee of the University of Cape Town Faculty of Health Sciences, HREC 726/2019. Written informed consent was obtained from all participants for inclusion in the study.

### Statistical analysis

Baseline descriptive statistics were used to characterise the study population. Percentages (%) and their 95% confidence intervals (95% CI) were used to depict proportions of categorical variables, while medians and interquartile ranges (IQR), or means with standard deviations (SD) summarised all continuous variables, as appropriate. The strength of association between two categorical variables was tested for using the χ2 or Fisher’s exact test. The Mann-Whitney U test was used to assess strength of association between continuous variables.

Potential risk factors for PCR confirmed RSV on IS were assessed. The variables assessed included age group of the patient, HIV and nutritional status, crèche attendance, household cigarette smoking, and history of breastfeeding. Relative risk was used to quantify the measure of effect between the RSV negative and RSV positive groups with respect to risk factors. Further analysis assessed the association between presence of RSV and presence of other respiratory pathogens in the IS sample using univariate and multivariable modelling. In all instances, generalised linear modelling using Poisson regression with robust error variance estimated adjusted relative risks (aRR) and their 95% confidence intervals. A significance level of P<0.05 was used for all analyses.

While the study analysed all 460 participants in describing the cohort and overall prevalence of confirmed RSV, assessment of risk and co-infections was limited to induced sputum samples. For the assessment of risk and co-infection IS sample with the 135 RSV cases detected on IS were used in the analysis. We assumed for the purpose of this study that RSV found in the IS (which accounted for 95% of cases) was most indicative of lower respiratory tract involvement. Other studies have also noted the challenges of determining pneumonia aetiology using specimens obtained from the upper respiratory tract. Whereas induced sputum allows for the expulsion of expectorated sputum from below the vocal cords (i.e., the ‘lower respiratory tract’), other procedures such as NP swabs are thought to collect organisms present in the upper respiratory tract. A positive finding from the upper respiratory tract may indicate a live pathogen, an infection or colonisation of the upper tract only or residual DNA from a previous infection. It is therefore difficult to prove causality of lung disease from the detection of an organism in the upper respiratory tract. Hence, we assumed for the purpose of this study that RSV found in the IS was most indicative of lower respiratory tract involvement [20–22].

## Results

### Baseline characteristics

The median age of the 460 children included was 7.8 (IQR 3.6–18.0) months and 258 (56.1%) were male. HIV status was determined to be HUU in 349 (75.9%), HEU in 92 (20.0%), whilst 19 (4.1%) were HIV infected. Of the HIV infected children, nine (47.4%) were on antiretroviral therapy (ART) with four having attained viral suppression. Immunisation status was known in all but nine of the enrolled children. Of the 451 with documented immunisation records, 325 (72.1%) had received immunisations appropriate for age at the time of admission. Nutrition status was recorded in all children with 415 (90.2%) children having normal weight-for-age (NWFA) and 45 (9.8%) children being classified as mildly to severely underweight-for-age (UWFA) **Table 1.**

**Table 1:** Baseline characteristics of the study children.

Baseline characteristic	Total number	RSV positive	RSV negative
	(N = 460)	(n = 142)	(n = 318)
	n (%)	n (%)	n (%)
** **Age** **			
< 3 months	86 (18.7)	43 (30.2)	43 (13.5)
3–11 months	203 (44.1)	68 (47.9)	135 (42.4)
12–23 months	94 (20.4)	22 (15.5)	72 (22.6)
> 23 months	77 (16.7)	9 (6.3)	68 (21.3)
** **Sex** **			
Male	258 (56)	84 (59.2)	174 (54.7)
Female	202 (43.9)	58 (40.8)	144 (45.2)
** **HIV status** **			
Unexposed uninfected	349 (75.9)	108 (76.1)	241 (75.8)
Exposed uninfected	92 (20.0)	32 (22.5)	60 (18.9)
Infected	19 (4.1)	2 (1.4)	17 (5.3)
** **Nutritional status** **			
Normal	351 (76.3)	116 (81.6)	235 (73.4)
Mild under-nutrition	64 (13.9)	19 (13.3)	45 (14.2)
Moderate under-nutrition	33 (7.2)	7 (4.9)	26 (8.2)
Severe under-nutrition	12 (2.6)	0 (0)	12 (3.8)
** **Immunisations appropriate for age** **			
Up to date for age	325 (70.7)	104 (73.2)	221 (69.5)
Not up to date for age	126 (27.4))	37 (26.1)	89 (28.0)
Unknown	9 (2.0)	1 (0.7)	8 (2.5)
** **Home cigarette smoking** **			
No home smoker	298 (64.8)	88 (61.0)	210 (66.0)
Home smoker	162 (35.2)	54 (38.0)	108 (34.0)
** **Creche attendance** **			
Creche attender	96 (20.9)	20 (14.1)	76 (23.9)
Non creche attender	364 (79.1)	122 (85.9)	242 (76.1)
** **History of breastfeeding** **			
Never breastfed	60 (13.0)	20 (14.1)	40 (12.6)
Breastfed first 4 months	323 (70.2)	103 (72.5)	220 (69.2)
Breastfed > 4 months	77 (16.7)	19 (13.3)	58 (18.2)

### Confirmed RSV infection

**Fig 2:**
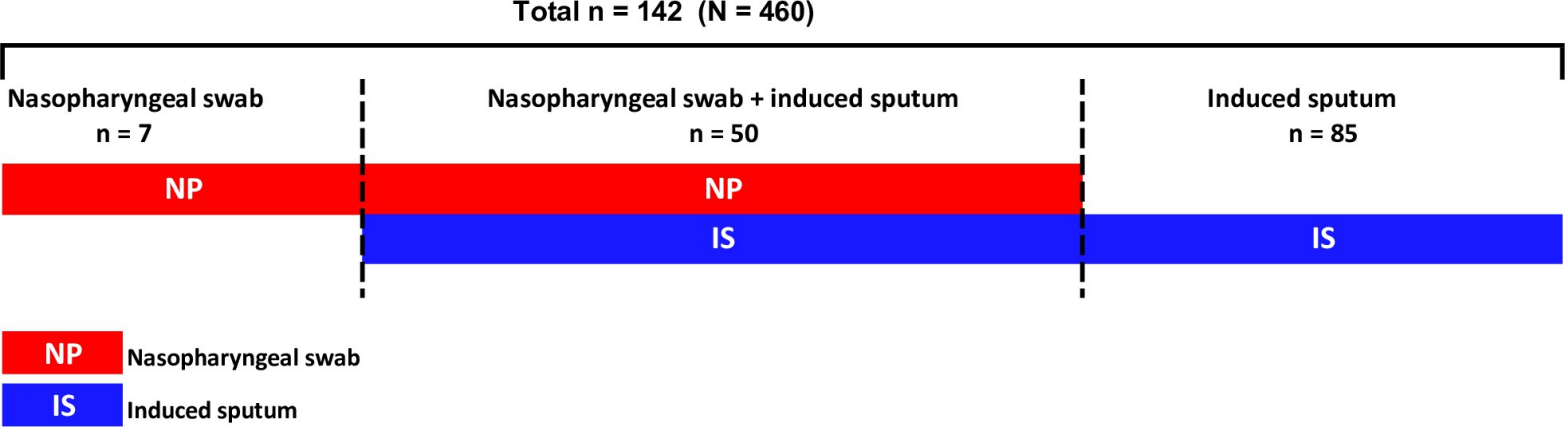
Number of respiratory syncytial virus (RSV) positive specimens by type of sample analysed.

PCR was positive for RSV in 142 (30.9%; 95%CI 26.7–35.3) of the 460 study participants. In 85 (59.9%) of the cases, PCR for RSV was detected on IS only, 7 cases (4.9%) were identified on NP swab only and 50 (35.2%) were identified on both NP and IS. **Fig 2**. Hence IS was able to identify 135 (95.1%) of the cases, while NP could identify 57 (40.1%) of the cases, P<0.001.

**Fig 3:**
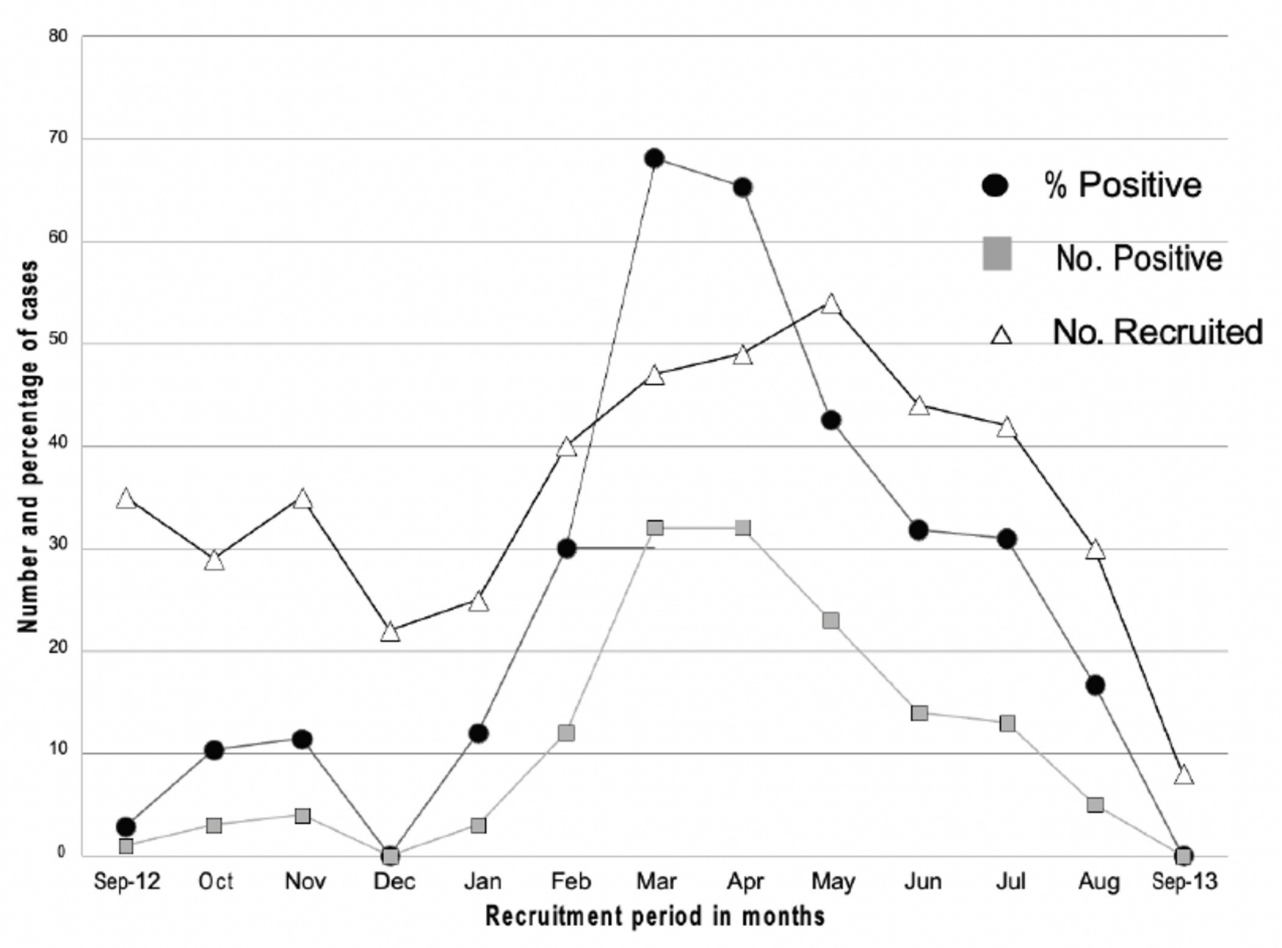
Percentage and number of confirmed respiratory syncytial virus cases per month.

Most RSV cases were seen in the months of February to July, with 126 (88.7%) of the 142 cases seen over this period. The peak of the cases was seen in the months of March and April. This observed pattern mirrors the known well-established pre-COVID-19 pandemic seasonal pattern of RSV in South Africa [23] **Fig 3.**

### Risk factors for confirmed RSV infection

**Fig 4:**
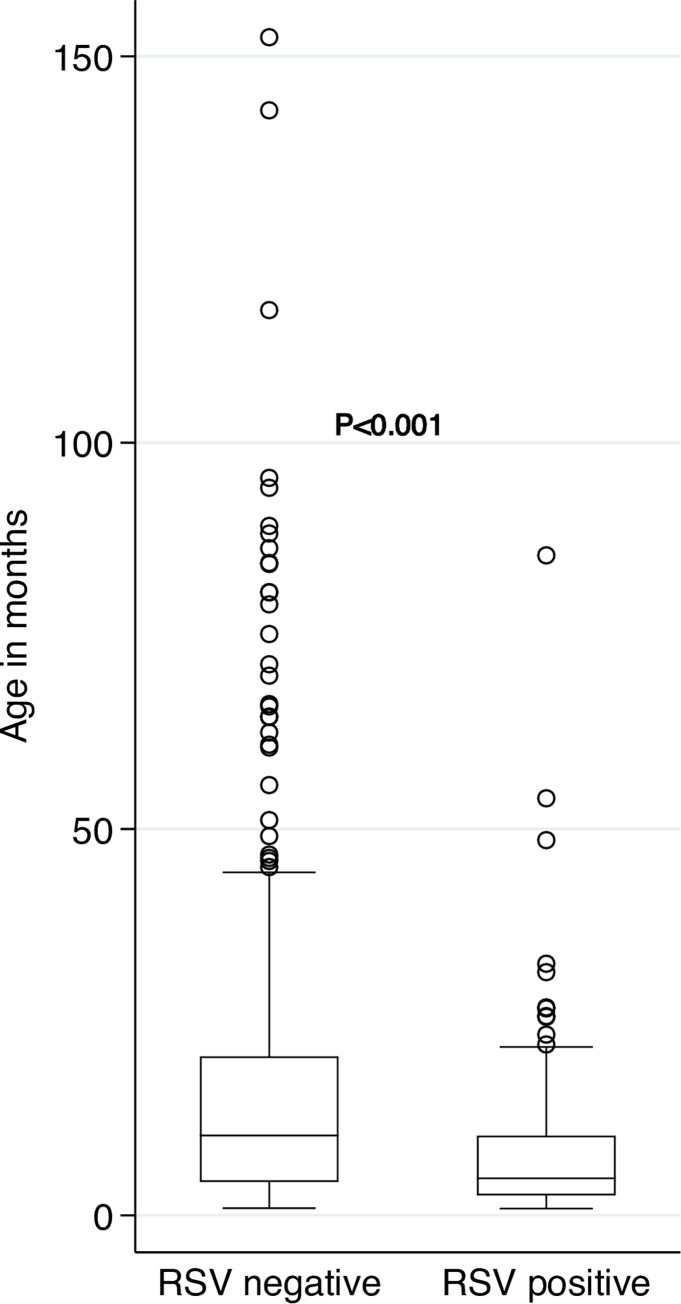
Ages of children by Respiratory syncytial virus (RSV) status.

The median age of children in whom RSV was detected on IS was 4.6 (IQR 2.4–9.7) months compared to children who were RSV-negative at 10.5 (IQR 4.4–21.3) months, P<0.001. **Fig 4**. PCR for RSV was positive in 82 (32.4%) of the 253 males and in 53 (26.3%) of the 201 females, P = 0.162.

The proportion of children testing positive for RSV was highest in those less than 3 months of age (43/86, 50.0%) compared to those over 23 months of age (9/77, 11.7%). The frequency of RSV decreased with an increase in age category. In 110 (31.6%) of the 348 children with normal weight-for-age, PCR for RSV was positive compared to 25 (26.3%) who were classified as mild-to-moderately UWFA. The risk of RSV was similar irrespective of HIV or crèche status, exposure to household tobacco smoke, or breastfeeding history **Table 2.**

**Table 2:** Risk factors for confirmed respiratory syncytial virus (RSV) infection in study children (N = 454).

		Relative Risk (95% Confidence Interval)	
Risk factor	Risk n/N (%)	Crude	Adjusted*
** **Age** **			
< 3 months	43/84 (51.2)	1	1
3–11 months	64/199 (32.2)	0.63 (0.47–0.84)	**0.63 (0.47–0.83)**
12–23 months	21/94 (22.3)	0.44 (0.28–0.67)	**0.44 (0.29–0.68)**
>23 months	7/77 (11.7)	0.18 (0.08–0.37)	**0.19 (0.09–0.39)**
** **Nutritional status** **			
Normal	110/348 (31.6)	1	1
Mild under-nutrition	18/62 (29.0)	0.92 (0.60–1.40)	0.93 (0.60–1.43)
Moderate under-nutrition	7/33 (21.2)	0.67 (0.34–1.32)	0.67 (0.35–1.32)
Severe under-nutrition	0	NA	NA
** **HIV status** **			
Unexposed uninfected	103/347 (29.7)	1	1
Exposed uninfected	30/88 (34.1)	1.15 (0.82–1.60)	1.12 (0.80–1.53)
Infected	2/19 (10.5)	0.36 (0.95–1.33)	0.48 (0.12–1.89)
** **Creche attendance** **			
Non attender	113/357 (31.7)	1	1
Attender	20/95 (21.1)	0.67 (0.44–1.01)	1.00 (0.67–1.52)
** **Breastfeeding history** **			
Never breastfed	19/58 (32.8)	1	1
Breastfed first 4 months	98/320 (30.6)	0.94 (0.62–1.40)	0.86 (0.56–1.31)
Breastfed > 4 months	18/76 (23.7)	0.72 (0.42–1.25)	0.64 (0.36–1.12)
** **Home cigarette smoking** **			
No home smoker	83/294 (28.2)	1	1
Home smoker	52/160 (32.5)	1.15 (0.86–1.54)	1.15 (0.87–1.51)

Risk ratio 95% confidence Intervals that do not cross the null value of 1 are shown in **bold typeface**

* Model adjusted for age, sex and HIV status

### Co-infection of RSV with other respiratory pathogens

**Fig 5:**
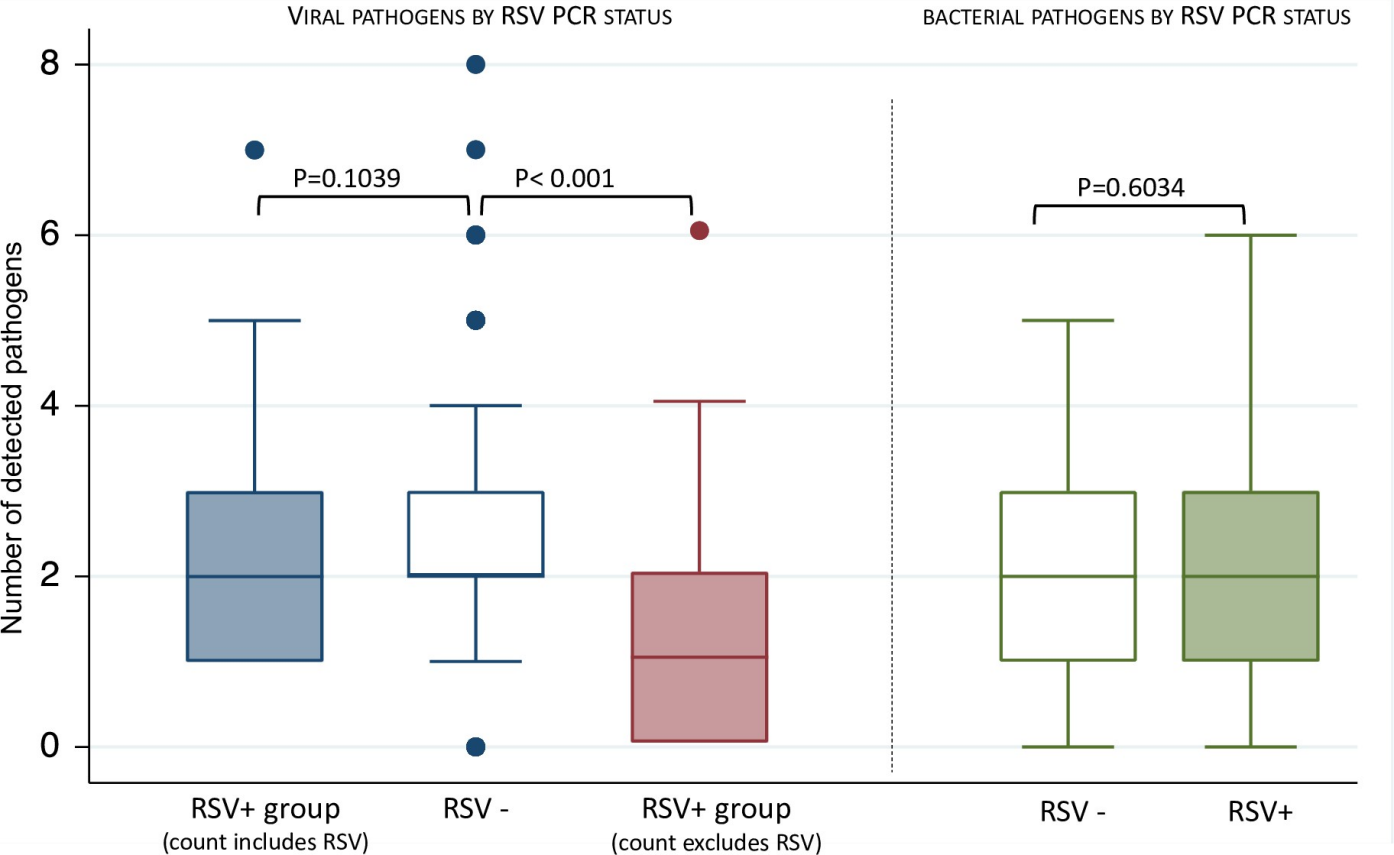
Number of pathogens detected on polymerase chain reaction (PCR) by Respiratory syncytial virus (RSV) status. NB: RSV positive group analysed with and without RSV included in the total count of viral pathogens.

The median number of detected viral pathogens was 2 (IQR 1–3) in RSV-positive children when RSV was included in the count compared to 2 (IQR 2–3) in RSV-negative children, P = 0.104. When compared to the latter, the median number of viral pathogens in RSV-positive children when RSV was excluded was 1 (IQR 0–2), P<0.001. Bacterial co-infections were found in 131 (97.0%) of the 135 RSV-positive and 304 (95.3%) of 318 RSV-negative cases, P = 0.608. The median number of bacterial pathogens in RSV-positive and RSV-negative children was 2 (IQR1-3) and 2 (IQR1-3) respectively, P = 0.603 **Fig 5.**

Rhinovirus was the most commonly detected virus in 34/135 (25.2%) of RSV-positive children while *Moraxella catarrhalis* was the most commonly found bacteria in 87/135 (64.4%). Overall, the frequency of other viruses was inversely associated with the presence of RSV, while the frequency of bacterial pathogens was similar in both groups of children **Table 3**.

**Table 3:** Presence of co-pathogens by RSV status.

		*RSV* PCR n (%)		
Pathogen ^¶^	Total n = 454 n (%)	Positive n = 135 n (%)	Negative n = 319 n (%)	P value^#^
** **Viral organisms** **				
**Rhinovirus**	222 (48.9)	34 (25.2)	188 (58.9)	**<0.001**
**Adenovirus**	117 (25.8)	9 (6.7)	108 (33.9)	**<0.001**
**Enterovirus-parechovirus**	80 (17.6)	9 (6.7)	71 (22.3)	**<0.001**
**Bocavirus**	80 (17.6)	20 (14.8)	60 (18.8)	0.347
**Parainfluenza (1,2,3 4)**	75 (16.5)	11 (8.1)	64 (20.1)	**<0.001**
**Metapneumovirus A & B**	45 (9.9)	2 (1.5)	43 (13.5)	**<0.001**
**Coronavirus**	35 (7.7)	11 (8.1)	24 (7.5)	0.848
**Influenza (A, B, C)**	28 (6.2)	4 (3.0)	24 (7.5)	**0.086**
** **Bacterial organisms** **				
***Moraxella catarrhalis***	295 (65.0)	87 (64.4)	208 (65.2)	0.914
***Streptococcus pneumoniae***	240 (52.9)	72 (53.3)	168 (52.7)	0.918
***Haemophilus influenzae***	231 (50.9)	61 (45.1)	170 (53.3)	0.124
***Staphylococcus aureus***	136 (30.0)	46 (34.0)	90 (28.2)	0.219
***Haemophilus influenzae B***	16 (3.5)	5 (3.7)	11 (3.4)	1.000
***Mycoplasma pneumoniae***	10 (2.2)	4 (3.0)	6 (1.9)	0.493
***Bordetella pertussis***	9 (2.0)	1 (0.74)	8 (2.5)	0.291
***Chlamydia pneumoniae***	7 (1.2)	2 (1.5)	5 (1.6)	1.000

IS = Induced sputum. # Two-sided Fisher’s exact or Chi Square tests P-values; Bold Typeface = P < 0.1

^**¶**^ Organisms shown in descending order of total frequency for each pathogen group

The presence of RSV was independently inversely associated with most viruses tested for on PCR after adjusting for potential confounding. **Table 4** shows relative risk of viral pathogens associated with RSV PCR status at P<0.1 after adjusting for potential confounding.

**Table 4:** Risk of viral pathogens associated with respiratory syncytial virus (RSV) PCR status at P<0.1.

Co-infection	*RSV* PCR n (%)		RR (95% Confidence interval)	
	Positive n = 135	Negative n = 319	. Crude	Adjusted^#^.
Rhinovirus	34 (25.2)	188 (58.9)	0.35 (0.25–0.50)	**0.40 (0.29–0.57)**
Adenovirus	9 (6.7)	108 (33.9)	0.21 (0.11–0.39)	**0.24 (0.13–0.45)**
Enterovirus-parechovirus	9 (6.7)	71 (22.3)	0.33 (0.18–0.63)	**0.39 (0.21–0.75)**
Parainfluenza	11 (8.1)	64 (20.1)	0.45 (0.25–0.79)	**0.44 (0.25–0.77)**
Metapneumovirus	2 (1.5)	43 (13.5)	0.14 (0.03–0.53)	**0.13 (0.03–0.51)**
Influenza (A, B, C)	4 (3.0)	24 (7.5)	0.46 (0.19–1.17)	**0.48 (0.19–1.17)**

RR = Relative risk

^#^ Multivariable model adjusted for age in months, sex and HIV status and nutritional status; confidence intervals not overlapping the null value of 1 are shown in bold typeface.

### Outcomes

None of the children with LRTI included in the study died during the study period.

Supplemental oxygen was required in 22 (16.3%) of the 135 children with RSV compared to 49 (15.4%) in the non-RSV group, P = 0.958. Length of hospitalisation in the RSV group averaged 3.3 (SD 4.2) days while those in the non-RSV group stayed an average of 2.7 (SD 3.3) days, P<0.001. Admission to a high care unit or ICU was required by 3 (2.2%) of the children in the RSV group compared with 9 (2.8%) children in the non- RSV group, P = 0.5.

## Discussion

This study has demonstrated that RSV is a common finding in children hospitalised with LRTI in Cape Town, South Africa. A third of participants in our study had laboratory-confirmed RSV. RSV showed a seasonal pattern and was more frequently detected in younger children in the cohort. Our study indicated that IS was the sample with a higher detection rate for RSV compared to NP. The presence of RSV was significantly associated with lower frequencies of other viruses. Other than age, no risk factor for RSV infection was identified. The presence of RSV PCR was associated with an increase in the length of hospital stay for LRTI.

The prevalence of detected RSV PCR in our study increased with decreasing age with the majority of infections occurring in children aged less than one year. These findings echo that of the PERCH study, a multi-country case-control study of pneumonia aetiology in 4232 children below 5 years of age. PERCH identified RSV to be the most common pathogen overall in the aetiology of severe pneumonia with 31.1% of cases due to RSV, a similar finding to our study. Similarly, RSV infection in the PERCH study was significantly higher in infants compared to older children with the highest frequency between the ages of one and six months [2]. The Drakenstein Health Study, a multi-year South African birth cohort study, demonstrated a similarly high frequency with RSV the most identified organism occurring in 23% of LRTI cases. Again, the highest incidence of RSV occurred in children less than 6 months of age [20].

The majority of RSV-related disease in our study children occurred in otherwise healthy, well-nourished children, a finding seen worldwide. A study done including participants from other LMIC’s including Nigeria, Gambia and India found no link between childhood malnutrition and RSV disease. In these studies, malnourished children demonstrated less severe disease and less RSV deaths compared to well-nourished children. This has been postulated to be due to a weaker immune response to the virus in malnourished children [24].

A multi-site study involving South African public hospitals called the Severe Acute Respiratory Virus Program (SARI), a countrywide respiratory virus surveillance program reported that HIV infection led to a 3-5-fold increased risk of hospitalisation in RSV-associated LRTI [23]. This ‘viral watch’ program also found that the incidence of RSV LRTI was higher in HEU children compared to those who were HUU [25]. In contrast, the present study did not demonstrate similar findings. Other factors such as crèche attendance, household smoke exposure, and breastfeeding history seem to be associated with RSV infection. However, the small number of children living with HIV in our study makes it impossible to do a meaningful comparison.

Our study found an increased median length of stay in the RSV-positive group compared to the RSV-negative group. A study done in the United Kingdom over eight successive bronchiolitis seasons in infants < 6 months old had similar findings. Detection of RSV was significantly associated with an increased average length of stay, with an average 84 hour stay in the RSV-positive group compared to a 48 hour stay in the RSV-negative group, (P<0.001) [26].

The previously mentioned Drakenstein Child Health study reported a higher sensitivity for IS in the diagnosis of several organisms, including RSV, compared to NP specimens [20]. The present study found the use of IS to have a significantly higher yield of detection of RSV compared to NP. RSV was more than twice likely to be identified on IS with a 95% detection rate compared to 40% in NP specimens from the same children.

In our study the presence of RSV was independently associated with a reduction in the frequency of most viruses tested for on PCR. This observation was particularly strong for adenovirus, enterovirus, rhinovirus, parainfluenza and metapneumovirus (P<0.01). A recent review article reported a lower-than-expected rate of co-infection between RSV and other viruses. The authors hypothesised that one virus may competitively inhibit the replication of another virus [27]. If this hypothesis is correct, it could explain our findings that RSV may potentially inhibit replication of other viruses.

The post COVID-19 period has seen a number of countries, including South Africa, struggle with RSV outbreaks that threaten the stability of the health care system [13, 28]. This phenomenon together with the burden and epidemiology of RSV disease demonstrated in this study highlights the importance of recent announcements of a potentially effective vaccine against RSV. This vaccine is intended to protect young infants through transplacental antibody transfer by immunising pregnant women [29]. This follows disappointments following failure of a previous promising candidate to reach the market [30].

Our study, from a single site, excluded children who were admitted directly to a high care or ICU setting. This may have affected our data report on disease severity, length of hospital stay and need for further respiratory support. In addition, the design of our study likely underestimated the burden of RSV disease. As only hospitalised cases were included, we missed a large number of children with respiratory disease seen in the outpatients’ section of the hospital during the same period–a group likely to constitute the bulk of RSV-infected children. The exclusion of prematurity and underlying cardiopulmonary disease as risk factors in the study participants is a limitation of this study. However, with the use of RSV PCR we were able to discover the large burden of RSV disease in our setting, especially in younger children and how IS may be a more useful tool in RSV diagnosis than NP. This study done pre-COVID-19 will provide a valuable comparison for any future similar studies considered in the now ‘post COVID’ era with respect to any changes in the RSV epidemiology in our area. Through all these findings we can better understand the trends in the burden of RSV disease in our setting and hope this may be used to prioritise interventions in the future.

## Conclusion

RSV is common in South African children hospitalised with LRTI and tends to affect younger children who require prolonged hospitalisation. Detection of RSV can potentially be improved by the use of IS specimens. Ultimately, there is an urgent need to find an effective vaccine to prevent RSV pneumonia in children, especially young infants. Once available such intervention should be made accessible to include children in LMICs that carry the greatest burden of RSV disease.

## Supporting information

S1 File Minimal dataset for multivariable analysis. (PDF)

## Abbreviations

ART: Antiretroviral treatment
COVID-19: SARS-CoV-2 disease
ELISA: Enzyme-linked immunosorbent assay
HEU: HIV exposed uninfected
HIV: Human immunodeficiency virus
HUU: HIV unexposed uninfected
ICU: Intensive care unit
IS: Induced sputum
LMIC: Low- and middle-income country
LRTI: Lower respiratory tract infection
NP: Nasopharyngeal swab
NWFA: Normal-weight-for-age
PCR: Polymerase chain reaction
RCWMCH: Red Cross War Memorial Children’s Hospital
RSV: Respiratory syncytial virus
UWFA: Underweight-for-age
WHO: World Health Organization
