# Supplementary material for: RSV infection in children hospitalised with severe lower respiratory tract infection in a low-middle-income setting: A cross-sectional observational study
Source: PLoS One. 2023 Sep 14;18(9):e0291433. doi: 10.1371/journal.pone.0291433 (PMC10501652; doi:10.1371/journal.pone.0291433)
Supplement: S1 File — (PDF) [file pone.0291433.s001.pdf]

MINIMUM DATA-SET - SUPPLEMENTARY MATERIAL

This minimal dataset includes unadjusted (Black text) and adjusted (Blue text) risk ratios for RSV and for co-detection of other pathogens using a Poisson-based general linear multivariable models to estimate the effect.

NB. The rest of the data is made fully and directly available within the manuscript in the form of:

- Text
- Tables
- Figures

The limited dataset shows the models used in the manuscript in their final fit

A. Factors associated with risk of detectable RSV

```
.
1 . *Age groups

. xi:glm rsv_is i.agegrp, fam(poisson) link(log) eform nolog robust
  i.agegrp          _Iagegrp_1-4        (naturally coded; _Iagegrp_1 omitted)

  Generalized linear models                      Number of obs   =        454
  Optimization     : ML                          Residual df     =        450
                                                 Scale parameter =          1
  Deviance         =  299.3119899                (1/df) Deviance =   .6651378
  Pearson          =  318.9999921                (1/df) Pearson  =   .7088889

  Variance function: V(u) = u                    [Poisson]
  Link function    : g(u) = ln(u)                [Log]

                                                 AIC             =   1.271612
  Log pseudolikelihood =  -284.655995            BIC             =  -2453.832
```

rsv_is	IRR	Robust Std. Err.	z	P>|z|	[95% Conf.	Interval]
_Iagegrp_2	.6282576	.0931844	-3.13	0.002	.4697707	.8402133
_Iagegrp_3	.4364176	.09605	-3.77	0.000	.2835068	.6718016
_Iagegrp_4	.1775899	.0668108	-4.59	0.000	.084955	.3712338
_cons	.5119048	.0545992	-6.28	0.000	.4153374	.6309244

Note: **_cons** estimates baseline incidence rate.

```
31 . xi:glm rsv_is  i.agegrp sex i.hivfinal, fam(poisson) link(log) eform nolog robust
   i.agegrp          _Iagegrp_1-4        (naturally coded; _Iagegrp_1 omitted)
   i.hivfinal        _Ihivfinal_1-3(naturally coded; _Ihivfinal_1 omitted)

   Generalized linear models                        Number  of  obs     =      454
   Optimization: ML                                 Residual  df        =      447
                                                    Scale  parameter  =          1
   Deviance          =   296.1900783                (1/df)  Deviance =.6626176
   Pearson           =  328.3814682                 (1/df)  Pearson  =.7346342

   Variance function: V(u) = u                      [Poisson]
   Link function: g(u) = ln(u)                      [Log]

                                                    AIC             =1.277952
   Log pseudolikelihood = -283.0950392              BIC             =  -2438.599
```

rsv_is	IRR	Robust Std. Err.	z	P>|z|	[95% Conf.	Interval]
_Iagegrp_2	.62534	.0919624	-3.19	0.001	.4687472	.8342452
_Iagegrp_3	.4393381	.0965718	-3.74	0.000	.2855582	.6759323
_Iagegrp_4	.1856247	.0697206	-4.48	0.000	.0889047	.3875669
sex	.8607523	.1218266	-1.06	0.289	.6522339	1.135934
_Ihivfinal_2	1.069855	.1785279	0.40	0.686	.7714073	1.483767
_Ihivfinal_3	.4179378	.2974417	-1.23	0.220	.1035907	1.686174
_cons	.547501	.0690551	-4.78	0.000	.4275873	.7010437

Note: **_cons** estimates baseline incidence rate.

```
32 . *Nutrition

33 . xi:glm rsv_is  i.anthro1, fam(poisson) link(log) eform nolog robust
   i.anthro1         _Ianthro1_1-4       (naturally coded; _Ianthro1_1 omitted)

   Generalized linear models                        Number of obs     =        454
   Optimization      : ML                           Residual df       =        450
                                                    Scale parameter =            1
   Deviance          =  319.6106863                 (1/df) Deviance =      .710246
   Pearson           =  307.9912627                 (1/df) Pearson  =      .684425

   Variance function: V(u) = u                      [Poisson]
   Link function     : g(u) = ln(u)                 [Log]

                                                    AIC             =    1.316323
   Log pseudolikelihood = -294.8053431              BIC             =   -2433.533
```

rsv_is	IRR	Robust Std. Err.	z	P>|z|	[95% Conf.	Interval]
_Ianthro1_2	.9184797	.1964388	-0.40	0.691	.6039762	1.396752
_Ianthro1_3	.6711021	.231524	-1.16	0.248	.3412966	1.319609
_Ianthro1_4	3.91e-07	1.22e-07	-47.29	0.000	2.12e-07	7.21e-07
_cons	.3160996	.0249514	-14.59	0.000	.270791	.3689893

Note: **_cons** estimates baseline incidence rate.

```
34 . xi:glm rsv_is  i.anthro1 i.hivfinal sex agemonth i.breastfeed, fam(poisson) link(log) eform nolog robust
   i.anthro1         _Ianthro1_1-4       (naturally coded; _Ianthro1_1 omitted)
   i.hivfinal        _Ihivfinal_1-3(naturally coded; _Ihivfinal_1 omitted)
   i.breastfeed      _Ibreastfee_1-3(naturally coded; _Ibreastfee_1 omitted)

   Generalized linear models                        Number  of  obs     =      454
   Optimization: ML                                 Residual  df        =      444
                                                    Scale  parameter  =          1
   Deviance          =   286.9019454                (1/df)  Deviance .6461756
   Pearson           =    362.10647                 (1/df)  Pearson  =.8155551
```

Variance function: **V(u) = u** [**Poisson**]
Link function: **g(u) = ln(u)** [**Log**]

AIC =**1.270709**
Log pseudolikelihood = **-278.4509727** BIC = **-2429.533**

rsv_is	IRR	Robust Std. Err.	z	P>|z|	[95% Conf.	Interval]
_Ianthro1_2	.924863	.2038186	-0.350.723		.6004711	1.424501
_Ianthro1_3	.6731041	.2300232	-1.160.247		.3445062	1.315126
_Ianthro1_4	1.56e-06	5.44e-07	-38.30.000		7.86e-07	3.09e-06
_Ihivfinal_2	1.019523	.1771797	0.10.911		.7252197	1.433259
_Ihivfinal_3	.564955	.3899189	-0.830.408		.146061	2.185212
sex	.8509265	.1193746	-1.150.250		.6463665	1.120225
agemonth	.9591454	.0114854	-3.480.000		.9368966	.9819226
_Ibreastfee_2	.8559385	.1846567	-0.720.471		.5607998	1.306403
_Ibreastfee_3	.6382666	.1831267	-1.560.118		.3637322	1.120011
_cons	.6343052	.1590411	-1.820.069		.3880378	1.036866

Note: **_cons** estimates baseline incidence rate.

35 . *HIV status

36 . xi:glm rsv_is i.hivfinal, fam(poisson) link(log) eform nolog robust
i.hivfinal _Ihivfinal_1-3 (naturally coded; _Ihivfinal_1 omitted)

Generalized linear models Number of obs = **454**
Optimization : **ML** Residual df = **451**
Scale parameter = **1**
Deviance = **323.7802663** (1/df) Deviance = **.7179163**
Pearson = **318.9999995** (1/df) Pearson = **.7073171**

Variance function: **V(u) = u** [**Poisson**]
Link function : **g(u) = ln(u)** [**Log**]

AIC = **1.321102**
Log pseudolikelihood = **-296.8901331** BIC = **-2435.482**

rsv_is	IRR	Robust Std. Err.	z	P>|z|	[95% Conf.	Interval]
_Ihivfinal_2	1.1485	.1951103	0.82	0.415	.8232398	1.602269
_Ihivfinal_3	.3546244	.2392593	-1.54	0.124	.0945098	1.330639
_cons	.29683	.0245526	-14.68	0.000	.252406	.3490727

Note: **_cons** estimates baseline incidence rate.

37 . xi:glm rsv_is i.hivfinal agemonth sex, fam(poisson) link(log) eform nolog robust
i.hivfinal _Ihivfinal_1-3(naturally coded; _Ihivfinal_1 omitted)

Generalized linear models Number of obs = **454**
Optimization: **ML** Residual df = **449**
Scale parameter = **1**
Deviance = **296.1797779** (1/df) Deviance .**6596432**
Pearson = **377.4127156** (1/df) Pearson =**.8405628**

Variance function: **V(u) = u** [**Poisson**]
Link function: **g(u) = ln(u)** [**Log**]

AIC =**1.269118**
Log pseudolikelihood = **-283.0898889** BIC = **-2450.846**

rsv_is	IRR	Robust Std. Err.	z	P>|z|	[95% Conf.	Interval]
_Ihivfinal_2	1.107987	.1831927	0.62	0.535	.8013058	1.532043
_Ihivfinal_3	.4771323	.3342285	-1.060.291		.1208863	1.883218

```
agemonth      |.9597864      .0117004    -3.370.001        .9371258     .9829949
          sex |.8465487      .1193423    -1.180.237        .6421754    1.115964
        _cons |.4902853      .0648069    -5.390.000        .3783863     .635276
-------------------------------------------------------------------------------
Note: _cons estimates baseline incidence rate.

38 . * Household cigarette smoker

39 . xi:glm rsv_is homesmoker, fam(poisson) link(log) eform nolog robust

   Generalized linear models                         Number of obs    =        454
   Optimization     : ML                             Residual df      =        452
                                                     Scale parameter =          1
   Deviance         =  326.8354305                   (1/df) Deviance =   .7230872
   Pearson          =          319                   (1/df) Pearson  =   .7057522

   Variance function: V(u) = u                       [Poisson]
   Link function    : g(u) = ln(u)                   [Log]

                                                     AIC              =   1.323426
   Log pseudolikelihood = -298.4177153               BIC              =  -2438.545

-------------------------------------------------------------------------------
             |               Robust
      rsv_is |        IRR   Std. Err.      z    P>|z|     [95% Conf. Interval]
-------------+-----------------------------------------------------------------
  homesmoker |   1.151205   .1694867     0.96   0.339     .8626488    1.536283
       _cons |   .2823129   .0262808   -13.59   0.000     .2352294    .3388207
-------------------------------------------------------------------------------
Note: _cons estimates baseline incidence rate.

40 . xi:glm rsv_is  homesmoker agemonth sex i.hivfinal, fam(poisson) link(log) eform nolog robust
   i.hivfinal       _Ihivfinal_1-3(naturally coded; _Ihivfinal_1 omitted)

   Generalized linear models                         Number  of  obs     =      454
   Optimization: ML                                  Residual  df        =      448
                                                     Scale  parameter   =         1
   Deviance         =      295.60271                 (1/df)  Deviance .6598275
   Pearson          =  385.8849725                   (1/df) Pearson  =.8613504

   Variance function: V(u) = u                       [Poisson]
   Link function: g(u) = ln(u)                       [Log]

                                                     AIC             =1.272253
   Log pseudolikelihood =   -282.801355              BIC              =  -2445.305

-------------------------------------------------------------------------------
             |               Robust
rsv_is       |        IRR   Std. Err.      z    P>|z|     [95% Conf. Interval]
-------------+-----------------------------------------------------------------
homesmoker   |1.144966      .1606163     0.97   0.335     .8697324    1.507299
agemonth     |.9594992      .0119256    -3.330.001        .9364079      .98316
          sex |.8518119       .119888     -1.10.254          .64646    1.122395
_Ihivfinal_2 |1.109672      .1824051      0.60.527        .8040371    1.531486
_Ihivfinal_3 |.4714082      .3329771    -1.060.287        .1180741    1.882087
       _cons |   .4662858   .0640921    -5.55   0.000      .356166    .6104525
-------------------------------------------------------------------------------
Note: _cons estimates baseline incidence rate.

41 . *Creche attendance

42 . xi:glm rsv_is  creche, fam(poisson) link(log) eform nolog robust

   Generalized linear models                         Number of obs    =        452
   Optimization     : ML                             Residual df      =        450
                                                     Scale parameter =          1
   Deviance         =  322.3044244                   (1/df) Deviance =   .7162321
   Pearson          =          319                   (1/df) Pearson  =   .7088889

   Variance function: V(u) = u                       [Poisson]
   Link function    : g(u) = ln(u)                   [Log]
```

```
                                                  AIC             =   1.310408
Log pseudolikelihood = -294.1522122               BIC             =  -2428.853
```

rsv_is	IRR	Robust Std. Err.	z	P>|z|	[95% Conf.	Interval]
creche	.6651141	.1420654	-1.91	0.056	.437606	1.010902
_cons	.3165266	.0246441	-14.77	0.000	.2717299	.3687083

Note: **_cons** estimates baseline incidence rate.

```
43 . xi:glm rsv_is  creche agemonth sex i.hivfinal, fam(poisson) link(log) eform nolog robust
i.hivfinal        _Ihivfinal_1-3(naturally coded; _Ihivfinal_1 omitted)

Generalized linear models                         Number  of  obs    =      452
Optimization: ML                                  Residual  df       =      446
                                                  Scale  parameter  =        1
Deviance          =   294.5983841                 (1/df)  Deviance .6605345
Pearson           =  376.7414159                  (1/df) Pearson  =.8447117

Variance function: V(u) = u                       [Poisson]
Link function: g(u)  = ln(u)                      [Log]

                                                  AIC             =1.266811
Log pseudolikelihood =  -280.299192               BIC             =  -2432.104
```

rsv_is	IRR	Robust Std. Err.	z	P>|z|	[95% Conf.	Interval]
creche	1.005761	.2132741	0.03	0.978	.663736	1.524032
agemonth	.9600256	.0122206	-3.20	0.001	.9363701	.9842788
sex	.8417905	.1199219	-1.2[illegible]	0.227	.6367097	1.112927
_Ihivfinal_2	1.121715	.186097	0.6[illegible]	0.489	.810335	1.552745
_Ihivfinal_3	.4831967	.3381987	-1.04	0.299	.1225608	1.905006
_cons	.4838815	.064263	-5.47	0.000	.3729866	.6277471

Note: **_cons** estimates baseline incidence rate.

```
44 . *History of breastfeeding

45 . xi:glm rsv_is  i.breastfeed, fam(poisson) link(log) eform nolog robust
i.breastfeed      _Ibreastfee_1-3     (naturally coded; _Ibreastfee_1 omitted)

Generalized linear models                         Number of obs   =        454
Optimization      : ML                            Residual df     =        451
                                                  Scale parameter =          1
Deviance          =  326.1984595                  (1/df) Deviance =   .7232782
Pearson           =          319                  (1/df) Pearson  =   .7073171

Variance function: V(u) = u                       [Poisson]
Link function     : g(u) = ln(u)                  [Log]

                                                  AIC             =   1.326428
Log pseudolikelihood = -298.0992298               BIC             =  -2433.063
```

rsv_is	IRR	Robust Std. Err.	z	P>|z|	[95% Conf.	Interval]
_Ibreastfee_2	.9348684	.1928709	-0.33	0.744	.623937	1.400749
_Ibreastfee_3	.7229917	.2018683	-1.16	0.245	.4182802	1.249682
_cons	.3275862	.0616944	-5.93	0.000	.2264741	.4738411

Note: **_cons** estimates baseline incidence rate.

```
46 . xi:glm rsv_is  i.breastfeed agemonth sex i.hivfinal i.anthro1, fam(poisson) link(log) eform nolog robust
i.breastfeed      _Ibreastfee_1-3     (naturally coded; _Ibreastfee_1 omitted)
i.hivfinal        _Ihivfinal_1-3      (naturally coded; _Ihivfinal_1 omitted)
```

i.anthro1 _Ianthro1_1-4 (naturally coded; _Ianthro1_1 omitted)

```
Generalized linear models                    Number  of  obs     =       454
Optimization: ML                             Residual  df        =       444
                                             Scale  parameter  =           1
Deviance          =   286.9019454            (1/df)  Deviance  = .6461756
Pearson           =    362.10647             (1/df) Pearson   =.8155551

Variance function: V(u) = u                  [Poisson]
Link function: g(u) = ln(u)                  [Log]

                                             AIC              =1.270709
Log pseudolikelihood = -278.4509727          BIC              =  -2429.533
```

rsv_is	IRR	Robust Std. Err.	z	P>|z|	[95% Conf.	Interval]
_Ibreastfee_2	.8559385	.1846567	-0.72	0.471	.5607998	1.306403
_Ibreastfee_3	.6382666	.1831267	-1.56	0.118	.3637322	1.120011
agemonth	.9591454	.0114854	-3.48	0.000	.9368966	.9819226
sex	.8509265	.1193746	-1.15	0.250	.6463665	1.120225
_Ihivfinal_2	1.019523	.1771797	0.11	0.911	.7252197	1.433259
_Ihivfinal_3	.564955	.3899189	-0.83	0.408	.146061	2.185212
_Ianthro1_2	.924863	.2038186	-0.35	0.723	.6004711	1.424501
_Ianthro1_3	.6731041	.2300232	-1.16	0.247	.3445062	1.315126
_Ianthro1_4	1.56e-06	5.44e-07	-38.33	0.000	7.86e-07	3.09e-06
_cons	.6343052	.1590411	-1.82	0.069	.3880378	1.036866

Note: **_cons** estimates baseline incidence rate.

B. Measure of effect of the strongly associated pathogens

```
82 . xi:glm rsv_is  adenovirus_is, fam(poisson) link(log) eform nolog robust

   Generalized linear models                       Number of obs     =        454
   Optimization     : ML                           Residual df       =        452
                                                   Scale parameter =            1
   Deviance         =  294.0869463                 (1/df) Deviance =    .6506348
   Pearson          =  318.9999413                 (1/df) Pearson  =    .7057521

   Variance function: V(u) = u                     [Poisson]
   Link function    : g(u) = ln(u)                 [Log]

                                                   AIC             =    1.251293
   Log pseudolikelihood = -282.0434732             BIC             =   -2471.293

   ------------------------------------------------------------------------------
                |               Robust
         rsv_is |        IRR   Std. Err.      z    P>|z|     [95% Conf. Interval]
   -------------+----------------------------------------------------------------
   adenovirus_is |   .2057388   .0675408    -4.82   0.000     .1081136    .3915185
          _cons |   .3738872   .0263852   -13.94   0.000     .3255902    .4293484
   ------------------------------------------------------------------------------
   Note: _cons estimates baseline incidence rate.

83 . xi:glm rsv_is adenovirus_is sex i.hivfinal agemonth i.anthro, fam(poisson) link(log) eform nolog robust
   i.hivfinal        _Ihivfinal_1-3      (naturally coded;
   i.anthro          _Ianthro_0hivfinal_1 omitted)(naturally coded;
                             _Ianthro_0 omitted)
   Generalized linear models                       Number  of  obs     =      454
   Optimization: ML                                Residual  df        =      447
                                                   Scale  parameter  =          1
   Deviance         =   268.4738621                (1/df)  Deviance .6006127
   Pearson          =   341.363646                 (1/df) Pearson   =.7636771

   Variance function: V(u) = u                     [Poisson]
   Link function: g(u) = ln(u)                     [Log]

                                                   AIC             =1.216903
   Log pseudolikelihood =  -269.236931             BIC             =  -2466.316

   ------------------------------------------------------------------------------
                |               Robust
         rsv_is |        IRR   Std. Err.      z    P>|z|     [95% Conf. Interval]
   -------------+----------------------------------------------------------------
   adenovirus_is |.2376718        .077267    -4.420.000     .1256761    .4494723
            sex |.7743422       .1036432     -1.90.056     .5956654    1.006615
   _Ihivfinal_2 |1.088574       .1678371      0.50.582      .804672     1.47264
   _Ihivfinal_3 |.5166705       .3513071    -0.970.331     .1362837    1.958769
   agemonth     |.9720664       .0086869    -3.170.002     .9551886    .9892424
   _Ianthro_1   |.5035036       .1775522    -1.950.052     .2522555    1.004997
          _cons |.5807465       .0643478    -4.900.000     .4673818    .7216081
   ------------------------------------------------------------------------------
   Note: _cons estimates baseline incidence rate.

84 . xi:glm rsv_is  enterovirusparechovirus_is, fam(poisson) link(log) eform nolog robust

   Generalized linear models                       Number of obs     =        454
   Optimization     : ML                           Residual df       =        452
                                                   Scale parameter =            1
   Deviance         =  313.4958574                 (1/df) Deviance =    .6935749
   Pearson          =   318.999999                 (1/df) Pearson  =    .7057522

   Variance function: V(u) = u                     [Poisson]
   Link function    : g(u) = ln(u)                 [Log]

                                                   AIC             =    1.294044
   Log pseudolikelihood = -291.7479287             BIC             =   -2451.884
```

rsv_is	IRR	Robust Std. Err.	z	P>|z|	[95% Conf.	Interval]
enterovirusparechovirus_is	.3339286	.1077421	-3.40	0.001	.1774244	.6284835
_cons	.3368984	.0244671	-14.98	0.000	.2922004	.3884339

Note: **_cons** estimates baseline incidence rate.

```
85 . xi:glm rsv_is  enterovirusparechovirus_is sex i.hivfinal agemonth i.anthro, fam(poisson) link(log) eform no
> log robust
i.hivfinal        _Ihivfinal_1-       (naturally coded; _Ihivfinal_1
i.anthro          3                   omitted)(naturally coded; _Ianthro_0
                  _Ianthro_0-1        omitted)
Generalized linear models                   Number  of  obs    =      454
Optimization: ML                            Residual  df       =      447
                                            Scale  parameter  =        1
Deviance          =     283.582883          (1/df)  Deviance .6344136
Pearson           =   354.6714608           (1/df) Pearson   =.7934485

Variance function: V(u) = u                 [Poisson]
Link function: g(u) = ln(u)                 [Log]

                                            AIC              =1.250183
Log pseudolikelihood = -276.7914415         BIC              =   -2451.207
```

rsv_is	IRR	Robust Std. Err.	z	P>|z|	[95% Conf.	Interval]
enterovirusparechovirus_i s	.3922153	.1297505	-2.830.005		.2050854	.7500915
sex	.872555	.1206441	-0.980.324		.6654281	1.144154
_Ihivfinal_2	1.148311	.1810443	0.880.380		.8430586	1.564087
_Ihivfinal_3	.4984869	.3441466	-1.010.313		.1288247	1.928894
agemonth	.9651369	.0106862	-3.200.001		.9444179	.9863104
_Ianthro_1	.5073216	.1811794	-1.900.057		.2519384	1.02158
_cons	.5305821	.0650613	-5.170.000		.4172305	.6747285

Note: **_cons** estimates baseline incidence rate.

```
86 . xi:glm rsv_is  influenza_is, fam(poisson) link(log) eform nolog robust

Generalized linear models                   Number of obs   =        454
Optimization      : ML                      Residual df     =        452
                                            Scale parameter =          1
Deviance          =  324.5286912            (1/df) Deviance =   .7179838
Pearson           =          319            (1/df) Pearson  =   .7057522

Variance function: V(u) = u                 [Poisson]
Link function     : g(u) = ln(u)            [Log]

                                            AIC             =   1.318345
Log pseudolikelihood = -297.2643456         BIC             =  -2440.851
```

rsv_is	IRR	Robust Std. Err.	z	P>|z|	[95% Conf.	Interval]
influenza_is	.4645583	.2179252	-1.63	0.102	.1852422	1.165039
_cons	.3075117	.0223826	-16.20	0.000	.2666281	.3546643

Note: **_cons** estimates baseline incidence rate.

```
87 . xi:glm rsv_is  influenza_is sex i.hivfinal agemonth i.anthro, fam(poisson) link(log) eform nolog robust
i.hivfinal        _Ihivfinal_1-3      (naturally coded;
i.anthro          _Ianthro_0-1ivfinal_1 omitted)(naturally coded;
                          _Ianthro_0 omitted)
Generalized linear models                   Number    of    obs        454
Optimization      : ML                      =   Residual    df         447
                                            =           Scale            1
Deviance          =  290.1232051            parameter =   (1/.6490452
                                            df) Deviance =
```

```
Pearson          =  366.4811457                (1/df) Pearson  =.8198683

Variance function: V(u) = u                    [Poisson]
Link function: g(u) = ln(u)                    [Log]

                                               AIC             =1.264589
Log pseudolikelihood = -280.0616026            BIC             =  -2444.666
```

rsv_is	IRR	Robust Std. Err.	z	P>|z|	[95% Conf.	Interval]
influenza_is	.4759774	.2189761	-1.610.107		.1931907	1.172699
sex	.8317374	.1159525	-1.30.186		.6328783	1.093081
_Ihivfinal_2	1.060954	.1720138	0.36.715		.7721298	1.457817
_Ihivfinal_3	.5270437	.3680705	-0.920.359		.1340895	2.071565
agemonth	.9601631	.0114272	-3.420.001		.9380255	.9828232
_Ianthro_1	.5192354	.183093	-1.860.063		.2601437	1.036371
_cons	.5366432	.0694605	-4.810.000		.4163995	.6916096

Note: **_cons** estimates baseline incidence rate.

```
88 . xi:glm rsv_is  parainfluenza_is, fam(poisson) link(log) eform nolog robust

Generalized linear models                      Number of obs   =        454
Optimization     : ML                          Residual df     =        452
                                               Scale parameter =          1
Deviance         =  319.3101931                (1/df) Deviance =   .7064385
Pearson          =  318.9999999                (1/df) Pearson  =   .7057522

Variance function: V(u) = u                    [Poisson]
Link function    : g(u) = ln(u)                [Log]

                                               AIC             =   1.306851
Log pseudolikelihood = -294.6550965            BIC             =   -2446.07
```

rsv_is	IRR	Robust Std. Err.	z	P>|z|	[95% Conf.	Interval]
parainfluenza_is	.4482796	.1292919	-2.78	0.005	.2547106	.7889525
_cons	.3271768	.0241269	-15.15	0.000	.2831475	.3780527

Note: **_cons** estimates baseline incidence rate.

```
89 . xi:glm rsv_is  parainfluenza_is sex i.hivfinal agemonth i.anthro, fam(poisson) link(log) eform nolog robust
i.hivfinal        _Ihivfinal_1-3      (naturally coded;
i.anthro          _Ianthro_0hivfinal_1 omitted)(naturally coded;
                          _Ianthro_0 omitted)
Generalized linear models                      Number  of  obs    =      454
Optimization: ML                               Residual  df       =      447
                                               Scale  parameter  =        1
Deviance         =   284.3153247               (1/df)  Deviance .6360522
Pearson          =  366.5863749                (1/df) Pearson  =.8201037

Variance function: V(u) = u                    [Poisson]
Link function: g(u) = ln(u)                    [Log]

                                               AIC             =1.251796
Log pseudolikelihood = -277.1576624            BIC             =  -2450.474
```

rsv_is	IRR	Robust Std. Err.	z	P>|z|	[95% Conf.	Interval]
parainfluenza_i	.4406798	.1260643	-2.860.004		.2515485	.7720129
s sex	.8660568	.1190129	-1.00.295		.6615685	1.133752
_Ihivfinal_2	1.105586	.1762883	0.60.529		.8088471	1.511188
	.504793	.3510827	-0.980.326		.1291529	1.972979
_Ihivfinal_3	.9599048	.0113244	-3.470.001		.937964	.9823588
agemonth	.545242	.1915873	-1.730.084		.2738381	1.085637
_Ianthro_1						

_cons	.5593961	.0708469	-4.590.000		.4364311	.7170067

Note: **_cons** estimates baseline incidence rate.

```
90 . xi:glm rsv_is  metapneumovirusaandb_is, fam(poisson) link(log) eform nolog robust

Generalized linear models                          Number of obs     =        454
Optimization     : ML                              Residual df       =        452
                                                   Scale parameter   =          1
Deviance         =  311.2694246                    (1/df) Deviance   =   .6886492
Pearson          =          319                    (1/df) Pearson    =   .7057522

Variance function: V(u) = u                        [Poisson]
Link function    : g(u) = ln(u)                    [Log]

                                                   AIC               =    1.28914
Log pseudolikelihood = -290.6347123                BIC               =  -2454.111
```

rsv_is	IRR	Robust Std. Err.	z	P>|z|	[95% Conf.	Interval]
metapneumovirusaandb_is	.136675	.0950769	-2.86	0.004	.0349589	.5343435
_cons	.3251834	.0231886	-15.75	0.000	.2827677	.3739615

Note: **_cons** estimates baseline incidence rate.

```
91 . xi:glm rsv_is  metapneumovirusaandb_is sex i.hivfinal agemonth i.anthro, fam(poisson) link(log) eform nolog
   >  robust
i.hivfinal         _Ihivfinal_1-       (naturally coded; _Ihivfinal_1
i.anthro           3                   omitted)(naturally coded; _Ianthro_0
                   _Ianthro_0-1        omitted)
Generalized linear models                          Number  of  obs    =     454
Optimization: ML                                   Residual  df       =     447
                                                   Scale  parameter   =       1
Deviance          =   275.1514655                  (1/df)  Deviance .6155514
Pearson           =  376.8445939                   (1/df) Pearson   =.8430528

Variance function: V(u) = u                        [Poisson]
Link function: g(u) = ln(u)                        [Log]

                                                   AIC              =1.231611
Log pseudolikelihood = -272.5757327                BIC              =  -2459.638
```

rsv_is	IRR	Robust Std. Err.	z P>|z|	[95% Conf.	Interval]
metapneumovirusaandb_i	.1279694	.0904274	-2.910.004	.0320345	.5112032
s	.8310559	.1129611	-1.30.173	.6366942	1.08475
sex	1.051131	.172398	0.30.761	.7621664	1.449653
_Ihivfinal_2	.4978475	.3496578	-0.990.321	.1256794	1.972098
_Ihivfinal_3	.9589935	.011443	-3.510.000	.936826	.9816856
agemonth	.5554381	.1924684	-1.700.090	.281631	1.095446
_Ianthro_1	.5775544	.0739769	-4.290.000	.4493301	.7423699
_cons					

Note: **_cons** estimates baseline incidence rate.

```
92 . xi:glm rsv_is  rhinovirus_is, fam(poisson) link(log) eform nolog robust

Generalized linear models                          Number of obs     =        454
Optimization     : ML                              Residual df       =        452
                                                   Scale parameter   =          1
Deviance         =   295.576151                    (1/df) Deviance   =   .6539295
Pearson          =  318.9999999                    (1/df) Pearson    =   .7057522

Variance function: V(u) = u                        [Poisson]
Link function    : g(u) = ln(u)                    [Log]

                                                   AIC               =   1.254573
Log pseudolikelihood = -282.7880755                BIC               =  -2469.804
```

rsv_is	IRR	Robust Std. Err.	z	P>|z|	[95% Conf.	Interval]
rhinovirus_is	.3517973	.0615044	-5.98	0.000	.249734	.4955727
_cons	.4353448	.0325869	-11.11	0.000	.3759398	.5041369

Note: _cons estimates baseline incidence rate.

```
93 . xi:glm rsv_is  rhinovirus_is sex i.hivfinal agemonth i.anthro, fam(poisson) link(log) eform nolog robust
i.hivfinal        _Ihivfinal_1-3      (naturally coded;
i.anthro          _Ianthro_0hivfinal_1 omitted)(naturally coded;
                          _Ianthro_0 omitted)
Generalized linear models                        Number of obs    =      454
Optimization: ML                                 Residual df      =      447
                                                 Scale  parameter =        1
Deviance          =      269.84855               (1/df)  Deviance .603688
Pearson           =   321.3058307                (1/df) Pearson  =.718805

Variance function: V(u) = u                      [Poisson]
Link function: g(u) = ln(u)                      [Log]

                                                 AIC             =1.219931
Log pseudolikelihood =  -269.924275              BIC             =  -2464.941
```

rsv_is	IRR	Robust Std. Err.	z	P>|z|	[95% Conf.	Interval]
rhinovirus_is	.4032936	.0706568	-5.180.000		.2860825	.5685274
sex	.8427485	.1112799	-1.30.195		.6505819	1.091677
_Ihivfinal_2	1.142427	.1766227	0.86.389		.8437821	1.546774
_Ihivfinal_3	.5009825	.3410861	-1.020.310		.1319154	1.90261
agemonth	.9674461	.0103705	-3.090.002		.9473323	.987987
_Ianthro_1	.5684825	.1975228	-1.630.104		.2877145	1.12324
_cons	.6524347	.0744661	-3.740.000		.5216564	.815999

Note: _cons estimates baseline incidence rate.
